# Hinge Atlas: relating protein sequence to sites of structural flexibility

**DOI:** 10.1186/1471-2105-8-167

**Published:** 2007-05-22

**Authors:** Samuel C Flores, Long J Lu, Julie Yang, Nicholas Carriero, Mark B Gerstein

**Affiliations:** 1 Department of Physics, Yale University, Bass 432, 266 Whitney Ave., New Haven, CT, USA; 2 Department of Molecular Biophysics and Biochemistry, Yale University, Bass 432, 266 Whitney Ave., New Haven, CT, USA; 3 Department of Computer Science, Yale University, Bass 432, 266 Whitney Ave., New Haven, CT, USA; 4 Computational Biology and Bioinformatics Program, Yale University, Bass 432, 266 Whitney Ave., New Haven, CT, USA

## Abstract

**Background:**

Relating features of protein sequences to structural hinges is important for identifying domain boundaries, understanding structure-function relationships, and designing flexibility into proteins. Efforts in this field have been hampered by the lack of a proper dataset for studying characteristics of hinges.

**Results:**

Using the Molecular Motions Database we have created a Hinge Atlas of manually annotated hinges and a statistical formalism for calculating the enrichment of various types of residues in these hinges.

**Conclusion:**

We found various correlations between hinges and sequence features. Some of these are expected; for instance, we found that hinges tend to occur on the surface and in coils and turns and to be enriched with small and hydrophilic residues. Others are less obvious and intuitive. In particular, we found that hinges tend to coincide with active sites, but unlike the latter they are not at all conserved in evolution. We evaluate the potential for hinge prediction based on sequence.

Motions play an important role in catalysis and protein-ligand interactions. Hinge bending motions comprise the largest class of known motions. Therefore it is important to relate the hinge location to sequence features such as residue type, physicochemical class, secondary structure, solvent exposure, evolutionary conservation, and proximity to active sites. To do this, we first generated the Hinge Atlas, a set of protein motions with the hinge locations manually annotated, and then studied the coincidence of these features with the hinge location. We found that all of the features have bearing on the hinge location. Most interestingly, we found that hinges tend to occur at or near active sites and yet unlike the latter are not conserved. Less surprisingly, we found that hinge residues tend to be small, not hydrophobic or aliphatic, and occur in turns and random coils on the surface. A functional sequence based hinge predictor was made which uses some of the data generated in this study. The Hinge Atlas is made available to the community for further flexibility studies.

## Background

Motions play an essential role in catalysis and protein-ligand interactions. In particular, hinge bending motions account for 45% of motions in a representative set from the Database of Macromolecular Motions[1] comprising domain hinge motions (31% of the total) and fragment hinge motions (14%) [2-4]. Thus understanding fundamental aspects of hinge bending mechanisms may lead to an improved understanding of the relationship between structure and function.

There are three levels of hinge prediction. The easiest case occurs when the atomic coordinates are available for two or more conformations of a given protein. In this case it is possible to visually inspect the motion to determine the hinge location, as we have done here. The process can also be automated with various available packages, including FlexProt[5,6], Hingefind[7] and DynDom[8]. A much more difficult problem is that of predicting hinges when only one set of structural atomic coordinates is available. Several algorithms have been developed for this purpose [9-15]. The very hardest case occurs when the sequence is known but no atomic coordinates are available at all.

The problem of finding flexible hinges between rigid regions based on sequence is in some ways similar to the problem of finding domain boundaries, which can be flexible or inflexible. Although little work has been done on the former problem, several algorithms exist to address the latter. In one significant contribution, Nagarajan and Yona[16] analyzed multiple sequence alignments and were able to identify domains with some accuracy. Marsden et al[17] focused on the case of proteins with no significant sequence homology to well characterized proteins and found that predicted secondary structure contained information about domain boundaries. Jones et al. combined PUU[18], DETECTIVE[19], and DOMAK[20] to make a consensus-based domain boundary predictor[21]. Heger et al. [22] created the Automatic Domain Decomposition Algorithm (ADDA)[23] and associated online database[22]. Murzin et al. created the SCOP (Structural Classification of Proteins) database[24]. For purposes of the above algorithms and classifications, however, domains are defined as proteins or regions of proteins having a common evolutionary origin[22,24]; flexibility is not a consideration. Indeed most small and medium sized proteins, such as those prevalent in the Hinge Atlas, consist of a single domain[24]. Therefore the problem of finding flexible hinges is not solved by finding domain boundaries as defined for these methods. Schlessinger et al[25] developed a method to predict B-factors from sequence, but it is not clear that B-factors obtained in this way would yield accurate flexibility predictions[26,27]. In light of the limitations of existing methods, the prediction of domain hinges from sequence is considered an open problem[16].

In this article we focus on the characterization of these hinges based on sequence. To that end, we compiled the Hinge Atlas, a manually annotated dataset of hinge bending motions, as well as a separate computer annotated dataset, both available for further studies. The Hinge Atlas has several applications. First, the statistical properties of hinges can be studied (composition, sequence correlations, coincidence with active sites, etc). Second, it can be used to benchmark hinge prediction programs. Third, by homology hinge annotations could potentially be transferred to proteins where the existence and location of a hinge are unknown. Fourth, the annotations could conceivably be used in future protein motion prediction programs. The first application was of most interest to us in the current work.

Our molecular motions database serves a wide variety of purposes, helping investigators understand the motion characteristics of individual proteins, as well as statistical properties of large groups of motions. It is the ideal platform for the current study, since it contains over 19000 morphs. A morph is a set of atomic coordinates for two homologous protein structures (usually obtained experimentally), plus several structures which our morph server generates as interpolations between the two[9,28]. Our server displays these structures in succession as a "movie" which suggests a possible trajectory of motion between the two conformations. In this study we compiled two representative sets of morphs with hinge annotation: a computer annotated set and a manually annotated set, the Hinge Atlas. Using mostly the latter, we addressed the following questions:

1. Are certain residue types differentially represented in hinges?

2. Do certain pairs of amino acids coincide with hinges?

3. Can sequence be used to predict hinges?

4. Do hinges coincide with active sites?

5. Do hinges prefer certain secondary structural elements?

6. Do hinge residues share physicochemical or steric properties?

7. Are hinge residues conserved in evolution?

As our first task, we computed the rate of occurrence of each residue type in the Hinge Atlas. Certain amino acids were found to be differentially represented in hinges in a statistically significant fashion. We also investigated whether certain consecutive pairs of residues were differentially represented in hinges. In the course of the above, we observed that one of the overrepresented residues (serine) is potentially catalytic; this was the original motivation for question 4 above. To answer that question, we searched the Catalytic Sites Atlas (CSA)[29] for close homologs to the proteins in our dataset, and extracted the active site residue numbers from those proteins for comparison to the Hinge Atlas annotation.

Our next task was to investigate hinge coincidence with secondary structure. Hinges are generally believed to occur in disordered regions, but this belief has never been tested or quantified rigorously to our knowledge.

Following up on our finding that hinges coincide with active site residues, we went on to the question, are hinge residues more likely to be conserved than other residues, as active sites are? We ranked the residues by relative conservation and examined the differences between hinge and non-hinge residues.

Significant correlations between sequence features and hinges were found in the above analyses. We computed Hinge Indices for each of these which may be used to relate sequence features to flexibility. We then sought to determine what predictive value sequence might have on its own and whether various sequence features collectively could be used for prediction.

We first made a simple GOR (Garnier-Osguthorpe-Robson) [30,31]-like predictor. We computed the log-odds rate of occurrence for residues located at the -8 to +8 positions along the sequence in the training set. We used this table to make predictions on the test set and examined their predictive power.

As a second approach, we made a composite Hinge Index, which we call HingeSeq, from the Hinge Indices of each of the sequence features found to be the strongest indicators of flexibility. The statistical significance of this measure was computed much as for the individual sequence features. To show that the measure is predictive, we again divided the Hinge Atlas into training and test sets and recomputed the relevant Hinge Indices to include only training set data. We used the regenerated HingeSeq to predict hinges in the test set and generated a Receiver Operating Characteristic (ROC) curve.

As a final step, we examined MolMovDB as a whole to determine whether any particular database bias was in evidence. We also used resampling[32] to check for sampling artifacts in the Hinge Atlas. Lastly, we compared the Hinge Atlas to our computer annotated dataset. The resulting work provides insight into the composition, physicochemical properties, geometry, and evolution of hinge regions in proteins.

## Methods

### Preparation of computer annotated hinge dataset

Prior to generating the manually annotated Hinge Atlas, we used computational methods to generate a dataset of hinge residues for our statistical studies. We began by running FlexProt[5], a leading hinge identification tool, on all morphs (pairs of homologous protein structures) in the Database of Macromolecular Motions[1,2,4,9,28,33,34] FlexProt works by matching and structurally aligning fragments in one structure with corresponding fragments in the other. The goal is to find fragment pairs which (1) have minimal RMSD and (2) are maximal in size. The hinges are then reported as the boundaries separating those fragments. Goal (2) is equivalent to minimizing the number of these hinges. Since domains are never completely rigid, RMSD tends to grow with fragment size and therefore goal (1) is in conflict with goal (2). This conflict is dealt with by providing the user with a series of adjustable parameters, and further by reporting not one but several alternative hinge locations from which the user can choose. We used a combination of computer and manual culling to select those morphs for which the identified hinges met the following criteria:

1. Motion was domain wise, i.e. two or more domains could be observed moving approximately as rigid bodies with respect to each other.

2. The identified hinge was located in the flexible region connecting two rigid domains, rather than in the domains themselves.

3. The morph trajectory was sterically reasonable, i.e. chains were not broken in the attempt to interpolate motion.

We found that FlexProt's Maximal[35,36] RMSD (Root Mean Square Deviation) parameter had a strong effect on the results. Therefore when FlexProt gave visibly incorrect results for a given morph, we reran the program, systematically varying this parameter. If one of these runs gave sufficiently accurate results, the annotation for that morph was entered into the database. We discarded immediately those morphs that did not exhibit clear hinge bending motion. Lastly, we removed redundant morphs using nrdb90[37].

Note that the definition of a hinge given in the introduction allows for a hinge of zero length. FlexProt indeed often returned such hinges. To deal with this, in all cases one residue on each side of the hinge, was taken to also belong to the hinge. Thus most hinges are two residues long. At the end of this process, the computer annotated set contained 273 morphs.

As described, the computer annotation of hinges requires significant human intervention and the results were often debatable. Many of the hinge annotations differed slightly but visibly from the boundary between rigid domains, such that the backbone flexions that could account for the domain motion were not seen in the predicted hinge region. In other cases hinges were missed, and some annotations appeared where no hinge existed. The more flagrantly misannotated hinges were removed from the dataset, but making the manual culling too stringent would simply have resulted in a dataset too small to be statistically meaningful. For these reasons, the computer annotated dataset was not used in most of this work. Nonetheless, the computer annotated dataset is arguably more objective then the manually annotated set described below, and so is made available to the community.

To address the accuracy issues, we decided to generate a manually annotated set of hinges – the Hinge Atlas. To generate this set we first created the Hinge Annotation Tool which can also be used by the public as we will now explain.

### The Hinge Annotation Tool

The creation of publicly accessible tools for manual annotation of hinges involved significant changes to the morph page. The morph page is the primary point on MolMovDB[1] for analyzing single morphs. It is accessible from the "movies" page or through our search tool, both linked to or visible on our front page. Our server also provides a link to this page in an email sent to the submitter of each morph request. We added all of the new tools to the "Hinge Analysis" tab on this page. The first of these is the Hinge Annotation tool. Each of three rows of "arrow" buttons on this tool move a highlighted window of two residues along the protein chain, allowing the user to highlight up to three hinges in a protein. The "Show all" button then highlights all selected residues in the Jmol viewer window. Once the user is satisfied with the hinge selection, clicking "Submit" records this selection in the database. Once the morph page is regenerated, a "Show public hinge" button will be visible which, when clicked, highlights the selected residues. Lastly, the user can use a pointing device to reorient the protein in the Jmol window to his/her liking. A GIF image based on that view can be generated by clicking on the "color by domain" link. The animation will be rendered using VMD's[38] "new cartoon" style, with the identified hinge region and two rigid domains each colored distinctively. The hinge annotations made in this way persist in our database for visualization and use by others, until overwritten. With minor modification, these tools were used to generate the Hinge Atlas dataset of manually annotated hinges. The criteria we used for selection are described in the following section.

Highlighting the Hinge Atlas hinges (described below) on the animated morph movie is a matter of going to the morph page and clicking on the "Hinge Analysis" tab as above and clicking the "Show Hinge Atlas hinge" button. The annotated hinge location will be rendered in green spacefill style, which contrasts with the white trace used elsewhere in the protein.

### Construction of the Hinge Atlas

The tools described above answer only the *technical *question of how we annotated hinges. In this section we clarify the motivation for the Hinge Atlas and its applications and answer the *scientific *question of how we decided on the precise location of the hinge for each morph.

For each morph in the Hinge Atlas, we used the Hinge Annotation Tool as described to select the hinge location. Motivated in part by our long term goal of providing a resource that could be used in motion prediction work, and in part by a desire to deepen basic understanding of protein motion, we asked ourselves the following question:

Would it be possible to approximately reproduce the observed motion by allowing flexure at the hinge points but keeping the regions between hinges rigid?

In order for this question to be answered in the affirmative, the hinge selection should be the one to best meet the following criteria:

1. The φ, ψ, and α (effective α-carbon to α-carbon)[39] torsion angles of hinge residues may often (but not always) be larger than those of their neighbors.

2. Amino acids on either side of the hinge residues must be co-moving with their respective rigid regions.

3. Rotations of one of the rigid regions about the hinge region must not result in significant and irreconcilable steric clashes.

In order to use (1) as a useful guide to selecting the hinge location, we made use of the torsion angle charts and graphs in the *structure analysis tools *section on the morph page. However often large rotations of the main chain are induced by multiple cooperative torsions in the hinge, and these may be individually small, particularly in α-helices[40]. The usefulness of this flexibility measure is further limited by the frequent occurrence of large torsion angles which do not coincide with hinges[39]. Nonetheless, when the precise location of the hinge was otherwise unclear, torsion angles were often examined to help adjust the selection.

Criterion (2) is a *definition *of a hinge. Sometimes the hinge was slightly longer than others, and in those cases we added more residues to the hinge, up to a limit of about five residues in total. If the hinge was distributed over too many residues such that no one short stretch could be said to constitute the entire hinge, then the morph was discarded from the Hinge Atlas, since the motion was not hingelike. Criterion (3) is a practical *requirement *of a working hinge. If substantial flexure at points outside the hinge is required to avoid domain interpenetration, then the choice of hinge location is incorrect, or the motion is not hinge but rather shear or unclassifiable[40].

The next question was, how to select the morphs which would be annotated and included in the Hinge Atlas. The entire Database of Macromolecular Motions (MolMovDB)[1] with (at the time) over 17000 morphs, could clearly not all be annotated given limited manpower. Further, only a minority of morphs (albeit a large one) exhibited hinge bending motion, and even within this group much redundancy existed.

To address these issues and make the annotation work manageable, we first selected a nonredundant subset of the morphs in MolMovDB by aligning all sequences to NRDB90[37]. This reduced the dataset to 1000 morphs. This was more manageable, but still the set contained many proteins which did not exhibit hinge bending motions. Fortunately we found that the score output by FlexProt, normalized by dividing by the number of residues, provided an accurate measure of the degree to which a protein exhibited hinge bending. High scores, close to unity, indicated proteins more likely to exhibit hinge bending motion. Lower scores, below 0.9 or so, were very unlikely to do so. We sorted the 1000 nonredundant morphs by descending normalized flexprot score (described earlier) and annotated them in that order. Those proteins for which we could find hinges allowing a positive answer to the question above were annotated and added to the Hinge Atlas. Those proteins which did not exhibit hinge bending motion or for which no suitable hinge could be found were discarded. At the end of this culling and annotation effort, the Hinge Atlas contained 214 nonredundant annotated morphs. We also manually annotated a small set of specifically fragment (rather than domain) hinge bending motions which may be useful for some studies, described below.

### Availability of datasets

In the course of this study we compiled a number of sets of morphs which can be viewed on our online galleries listed and linked to on our sets page[41]. The Hinge Atlas and computer annotated sets are compared more rigorously in the "Statistical comparison of datasets" section. The galleries provide easy browsing and visual inspection of morph movies sharing certain characteristics. The sets offered include:

### Nonredundant

No two morphs in this set have more than 90% sequence homology. This set was compiled by alignment to proteins in nrdb90.

### Catalytic Sites Atlas

All morphs in this set have annotated active sites which can be highlighted in the jmol viewer.

### Catalytic Sites Atlas (nonredundant)

Same as above, but with redundant morphs removed by comparison to nrdb90.

### FlexProt Hinges

Computer annotated set used in parts of this study and described above. We consider it to be less useful than the Hinge Atlas, but the data is nonetheless made available.

### Fragment Hinge Motions

A small set of hinge bending motions involving fragments smaller than domains, as alluded to in the previous section.

### Hinge Atlas

Contains the manually annotated protein pairs used in this study. A link on the sets page permits the download of the sequence data (including residue number, residue type, hinge annotation, catalytic site annotation, and secondary structure) in mySQL format. The same data is available in tab-delimited text format which is human readable and importable into MS Excel and other packages. Another link on the same page facilitates the download of the interpolated structure files associated with each morph in the Hinge Atlas set.

Clicking on the thumbnail image leads to the "movies" page, where users can browse through the 214 proteins in the Hinge Atlas. Clicking on any of the protein thumbnail images, in turn, leads to the corresponding morph page, where the hinge annotation can be viewed as described in the "Hinge Annotation Tool" section

### Method for analyzing relative frequency of occurrence

Throughout this study, we will be comparing how often a particular entity (be it a certain amino acid, a certain pair of amino acids, a certain class of amino acids, a certain secondary structural element etc.) occurs in hinges versus everywhere in the Hinge Atlas or another of the datasets described above. The statistical analysis will be the same regardless of the particulars, so we will here present the general approach and later only mention adjustments particular to the specific question addressed.

First we defined the following variables:

*D *= total number of residues in the dataset

*H *= total number of residues in hinges in the dataset

*C *= classification scheme used to create groups of residue positions. For example, *C *could be secondary structure, degree of conservation, etc.

*c *= a particular grouping of residues, where *c *∈ *C*. For instance, if *C *= secondary structure, then *c *= *helix *is the class of all residues in helices, *c *= *strand *is the class of all residues in strands, etc. Another example might be *C *= evolutionary conservation, with *c *= *cons1 *= top 20% most conserved residues, *c *= *cons2 *= second 20% most conserved, etc.

*a*_*c *_= set of all residues of class *c *in the dataset.

*d*_*c *_= number of times residues of class *c *occurred *anywhere *in the dataset.

*h*_*c *_= number of times residues of a particular class *c *occurred in hinges.

These can be used to estimate various probabilities as follows:

*p*(*a*_*c*_) = *d*_*c*_/*D *is the prior probability of *c *– in other words, the probability that residues of class *c *occur anywhere in the dataset.

*p*(*a*_*c*_|*h*) = *h*_*c*_/*H *is the conditional probability that a residue belongs to class *c*, given it is a hinge.

A quantity that is of interest in hinge prediction is the posterior probability *p*(*h*|*a*_*c*_), the probability that a residue is a hinge given it is in *a*_*c*_. We obtain this from Bayes' rule:

p(h|ac)=p(ac|h)⋅p(h)p(ac)=hcdc
 MathType@MTEF@5@5@+=feaafiart1ev1aaatCvAUfKttLearuWrP9MDH5MBPbIqV92AaeXatLxBI9gBaebbnrfifHhDYfgasaacH8akY=wiFfYdH8Gipec8Eeeu0xXdbba9frFj0=OqFfea0dXdd9vqai=hGuQ8kuc9pgc9s8qqaq=dirpe0xb9q8qiLsFr0=vr0=vr0dc8meaabaqaciaacaGaaeqabaqabeGadaaakeaacqWGWbaCcqGGOaakcqWGObaAcqGG8baFcqWGHbqydaWgaaWcbaGaem4yamgabeaakiabcMcaPiabg2da9maalaaabaGaemiCaaNaeiikaGIaemyyae2aaSbaaSqaaiabdogaJbqabaGccqGG8baFcqWGObaAcqGGPaqkcqGHflY1cqWGWbaCcqGGOaakcqWGObaAcqGGPaqkaeaacqWGWbaCcqGGOaakcqWGHbqydaWgaaWcbaGaem4yamgabeaakiabcMcaPaaacqGH9aqpdaWcaaqaaiabdIgaOnaaBaaaleaacqWGJbWyaeqaaaGcbaGaemizaq2aaSbaaSqaaiabdogaJbqabaaaaaaa@52B3@

Equation 1

Where the prior probability that a residue is a hinge is given by p(h)=(HD)
 MathType@MTEF@5@5@+=feaafiart1ev1aaatCvAUfKttLearuWrP9MDH5MBPbIqV92AaeXatLxBI9gBaebbnrfifHhDYfgasaacH8akY=wiFfYdH8Gipec8Eeeu0xXdbba9frFj0=OqFfea0dXdd9vqai=hGuQ8kuc9pgc9s8qqaq=dirpe0xb9q8qiLsFr0=vr0=vr0dc8meaabaqaciaacaGaaeqabaqabeGadaaakeaacqWGWbaCcqGGOaakcqWGObaAcqGGPaqkcqGH9aqpcqGGOaakdaWcaaqaaiabdIeaibqaaiabdseaebaacqGGPaqkaaa@3612@.

We further define the hinge index *HI*, similar to the domain linker index used in Armadillo[42]:

HI(ac)=log⁡10p(ac|h)p(ac)
 MathType@MTEF@5@5@+=feaafiart1ev1aaatCvAUfKttLearuWrP9MDH5MBPbIqV92AaeXatLxBI9gBaebbnrfifHhDYfgasaacH8akY=wiFfYdH8Gipec8Eeeu0xXdbba9frFj0=OqFfea0dXdd9vqai=hGuQ8kuc9pgc9s8qqaq=dirpe0xb9q8qiLsFr0=vr0=vr0dc8meaabaqaciaacaGaaeqabaqabeGadaaakeaacqWGibascqWGjbqscqGGOaakcqWGHbqydaWgaaWcbaGaem4yamgabeaakiabcMcaPiabg2da9iGbcYgaSjabc+gaVjabcEgaNnaaBaaaleaacqaIXaqmcqaIWaamaeqaaOWaaSaaaeaacqWGWbaCcqGGOaakcqWGHbqydaWgaaWcbaGaem4yamgabeaakiabcYha8jabdIgaOjabcMcaPaqaaiabdchaWjabcIcaOiabdggaHnaaBaaaleaacqWGJbWyaeqaaOGaeiykaKcaaaaa@4959@

Equation 2

The argument of the log is the ratio of the observed frequency of occurrence of classes of amino acids *a*_*c *_in hinges, over the expected. Note that this argument is close to the likelihood ratio p(ac|h)p(ac|~h)
 MathType@MTEF@5@5@+=feaafiart1ev1aaatCvAUfKttLearuWrP9MDH5MBPbIqV92AaeXatLxBI9gBaebbnrfifHhDYfgasaacH8akY=wiFfYdH8Gipec8Eeeu0xXdbba9frFj0=OqFfea0dXdd9vqai=hGuQ8kuc9pgc9s8qqaq=dirpe0xb9q8qiLsFr0=vr0=vr0dc8meaabaqaciaacaGaaeqabaqabeGadaaakeaadaWcaaqaaiabdchaWjabcIcaOiabdggaHnaaBaaaleaacqWGJbWyaeqaaOGaeiiFaWNaemiAaGMaeiykaKcabaGaemiCaaNaeiikaGIaemyyae2aaSbaaSqaaiabdogaJbqabaGccqGG8baFcqGG+bGFcqWGObaAcqGGPaqkaaaaaa@3FC8@ used in Bayesian statistics[43] because *H *is so small compared to *D*. The quantity *HI *yields an intuitive measure of the enrichment of certain classes of residues in hinges, with positive numbers indicating enrichment and negative numbers indicating scarcity. Just because the *HI *is nonzero, however, does not mean that the differential representation has statistical significance. To establish the latter, we considered two statistical hypotheses:

*H*_0_: *The null hypothesis*.

Assume *h*_*c *_is a randomly distributed random variable with mean *μ*_*h*_. The null hypothesis states that:

μh=dcD⋅H
 MathType@MTEF@5@5@+=feaafiart1ev1aaatCvAUfKttLearuWrP9MDH5MBPbIqV92AaeXatLxBI9gBaebbnrfifHhDYfgasaacH8akY=wiFfYdH8Gipec8Eeeu0xXdbba9frFj0=OqFfea0dXdd9vqai=hGuQ8kuc9pgc9s8qqaq=dirpe0xb9q8qiLsFr0=vr0=vr0dc8meaabaqaciaacaGaaeqabaqabeGadaaakeaaiiGacqWF8oqBdaWgaaWcbaGaemiAaGgabeaakiabg2da9maalaaabaGaemizaq2aaSbaaSqaaiabdogaJbqabaaakeaacqWGebaraaGaeyyXICTaemisaGeaaa@3858@

If this is true, then the hinge set is chosen without replacement in an unbiased fashion from the dataset, and *p*(*a*_*c*_|*h*) is given by the hypergeometric distribution (Equation 3).

*H*_1_, *The alternate hypothesis*.

This states that:

μh≠dcD⋅H.
 MathType@MTEF@5@5@+=feaafiart1ev1aaatCvAUfKttLearuWrP9MDH5MBPbIqV92AaeXatLxBI9gBaebbnrfifHhDYfgasaacH8akY=wiFfYdH8Gipec8Eeeu0xXdbba9frFj0=OqFfea0dXdd9vqai=hGuQ8kuc9pgc9s8qqaq=dirpe0xb9q8qiLsFr0=vr0=vr0dc8meaabaqaciaacaGaaeqabaqabeGadaaakeaaiiGacqWF8oqBdaWgaaWcbaGaemiAaGgabeaakiabgcMi5oaalaaabaGaemizaq2aaSbaaSqaaiabdogaJbqabaaakeaacqWGebaraaGaeyyXICTaemisaGKaeiOla4caaa@39FD@

It is equivalent to saying that *p*(*a*_*c*_|*h*) is *not p*(*a*_*c*_), and therefore the null hypothesis can be rejected. We test this as follows. If it is the case that

hcH>dcD,
 MathType@MTEF@5@5@+=feaafiart1ev1aaatCvAUfKttLearuWrP9MDH5MBPbIqV92AaeXatLxBI9gBaebbnrfifHhDYfgasaacH8akY=wiFfYdH8Gipec8Eeeu0xXdbba9frFj0=OqFfea0dXdd9vqai=hGuQ8kuc9pgc9s8qqaq=dirpe0xb9q8qiLsFr0=vr0=vr0dc8meaabaqaciaacaGaaeqabaqabeGadaaakeaadaWcaaqaaiabdIgaOnaaBaaaleaacqWGJbWyaeqaaaGcbaGaemisaGeaaiabg6da+maalaaabaGaemizaq2aaSbaaSqaaiabdogaJbqabaaakeaacqWGebaraaGaeiilaWcaaa@3692@

and if we choose a significance threshold of 0.05, we can reject *H*_0 _iff our p-value ∑x=h(a)h∞HYP(H,D,x,dc)<0.05
 MathType@MTEF@5@5@+=feaafiart1ev1aaatCvAUfKttLearuWrP9MDH5MBPbIqV92AaeXatLxBI9gBaebbnrfifHhDYfgasaacH8akY=wiFfYdH8Gipec8Eeeu0xXdbba9frFj0=OqFfea0dXdd9vqai=hGuQ8kuc9pgc9s8qqaq=dirpe0xb9q8qiLsFr0=vr0=vr0dc8meaabaqaciaacaGaaeqabaqabeGadaaakeaadaaeWbqaaiabdIeaijabdMfazjabdcfaqjabcIcaOiabdIeaijabcYcaSiabdseaejabcYcaSiabdIha4jabcYcaSiabdsgaKnaaBaaaleaacqWGJbWyaeqaaOGaeiykaKIaeyipaWJaeGimaaJaeiOla4IaeGimaaJaeGynaudaleaacqWG4baEcqGH9aqpcqWGObaAcqGGOaakcqWGHbqycqGGPaqkdaWgaaadbaGaemiAaGgabeaaaSqaaiabg6HiLcqdcqGHris5aaaa@4BC9@. The left hand side of the latter inequality can be interpreted as the probability that *h*_*c *_or more residues of class *a*_*c *_could be found in hinges, assuming *H*_0 _and given *H*, *D*, and *d*_*c*_. The argument of the sum is the hypergeometric function, which gives the probability that *d*_*c *_residues taken without replacement from a set of *D *residues of which *H *are hinges, would contain exactly *x *hinges:

HYP(H,D,x,dc)(dcx)(D−dcH−x)(DH)
 MathType@MTEF@5@5@+=feaafiart1ev1aaatCvAUfKttLearuWrP9MDH5MBPbIqV92AaeXatLxBI9gBaebbnrfifHhDYfgasaacH8akY=wiFfYdH8Gipec8Eeeu0xXdbba9frFj0=OqFfea0dXdd9vqai=hGuQ8kuc9pgc9s8qqaq=dirpe0xb9q8qiLsFr0=vr0=vr0dc8meaabaqaciaacaGaaeqabaqabeGadaaakeaacqWGibascqWGzbqwcqWGqbaucqGGOaakcqWGibascqGGSaalcqWGebarcqGGSaalcqWG4baEcqGGSaalcqWGKbazdaWgaaWcbaGaem4yamgabeaakiabcMcaPmaalaaabaWaaeWaaeaafaqaaeGabaaabaGaemizaq2aaSbaaSqaaiabdogaJbqabaaakeaacqWG4baEaaaacaGLOaGaayzkaaWaaeWaaeaafaqaaeGabaaabaGaemiraqKaeyOeI0Iaemizaq2aaSbaaSqaaiabdogaJbqabaaakeaacqWGibascqGHsislcqWG4baEaaaacaGLOaGaayzkaaaabaWaaeWaaeaafaqaaeGabaaabaGaemiraqeabaGaemisaGeaaaGaayjkaiaawMcaaaaaaaa@4E8F@

Equation 3

Otherwise, if it is the case that

hcH<dcD,
 MathType@MTEF@5@5@+=feaafiart1ev1aaatCvAUfKttLearuWrP9MDH5MBPbIqV92AaeXatLxBI9gBaebbnrfifHhDYfgasaacH8akY=wiFfYdH8Gipec8Eeeu0xXdbba9frFj0=OqFfea0dXdd9vqai=hGuQ8kuc9pgc9s8qqaq=dirpe0xb9q8qiLsFr0=vr0=vr0dc8meaabaqaciaacaGaaeqabaqabeGadaaakeaadaWcaaqaaiabdIgaOnaaBaaaleaacqWGJbWyaeqaaaGcbaGaemisaGeaaiabgYda8maalaaabaGaemizaq2aaSbaaSqaaiabdogaJbqabaaakeaacqWGebaraaGaeiilaWcaaa@368E@

then we reject *H*_0 _iff our p-value ∑x=−∞h(a)HYP(H,D,x,d(ai))<0.05
 MathType@MTEF@5@5@+=feaafiart1ev1aaatCvAUfKttLearuWrP9MDH5MBPbIqV92AaeXatLxBI9gBaebbnrfifHhDYfgasaacH8akY=wiFfYdH8Gipec8Eeeu0xXdbba9frFj0=OqFfea0dXdd9vqai=hGuQ8kuc9pgc9s8qqaq=dirpe0xb9q8qiLsFr0=vr0=vr0dc8meaabaqaciaacaGaaeqabaqabeGadaaakeaadaaeWbqaaiabdIeaijabdMfazjabdcfaqjabcIcaOiabdIeaijabcYcaSiabdseaejabcYcaSiabdIha4jabcYcaSiabdsgaKjabcIcaOiabdggaHnaaBaaaleaacqWGPbqAaeqaaOGaeiykaKIaeiykaKIaeyipaWJaeGimaaJaeiOla4IaeGimaaJaeGynaudaleaacqWG4baEcqGH9aqpcqGHsislcqGHEisPaeaacqWGObaAcqGGOaakcqWGHbqycqGGPaqka0GaeyyeIuoaaaa@4E2E@.

## Results

### Are certain amino acids more likely to occur in hinges?

We applied the described statistical formalism to the problem of amino acid frequency of occurrence in hinges by taking *C *= amino acid type, and *c *to designate each of the 20 canonical amino acids. HI scores and p-values were thus calculated for each of 20 identifications of *c *corresponding to the 20 canonical amino acids.

**Figure 1:**
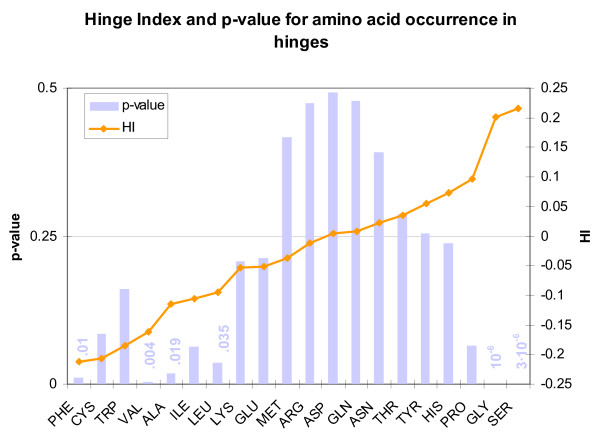
Amino acids arranged in ascending order of Hinge Index (HI) (orange line). Low p-values (vertical bars) indicate high statistical significance. Legend information applies to similar graphs in this work.

We found that glycine and serine are overrepresented in a highly significant fashion. We also found phenylalanine, valine, alanine, and leucine to be underrepresented, albeit with lower significance (Figure 1, Table 1). We also investigated the frequency of occurrence of sequential *pairs *of amino acids in hinges, but since 400 sequential pairs are possible the significance of the results was much lower and no conclusion could be drawn.

**Table 1:** Amino acid frequency of occurrence in hinges.

Residue	Occurrence in hinges	Occurrence everywhere	HI_amino acid_	p-value
ALA	56	4577	-0.114	0.019
ARG	40	2578	-0.011	0.475
ASN	39	2325	0.023	0.392
ASP	51	3173	0.004	0.492
CYS	9	909	-0.206	0.085
GLN	32	1970	0.009	0.479
GLU	51	3601	-0.051	0.213
GLY	108	4269	0.201	1·10^-6^
HIS	22	1166	0.074	0.238
ILE	38	3049	-0.106	0.064
LEU	62	4851	-0.095	0.035
LYS	48	3413	-0.054	0.207
MET	18	1229	-0.036	0.416
PHE	20	2051	-0.213	0.010
PRO	50	2513	0.097	0.064
SER	89	3401	0.216	3·10^-6^
THR	56	3248	0.035	0.287
TRP	7	672	-0.184	0.160
TYR	33	1829	0.054	0.255
VAL	44	4015	-0.162	0.004

Total	873	54839		

### Are residues within a certain distance of an active site more likely to be hinge residues?

As mentioned earlier, the fact that one of the overrepresented residues is potentially catalytic led us to suspect that hinge residues are more likely to occur in active sites, or within a few residues of an active site, than would be expected by chance. This would make sense from a biochemical and mechanical perspective. Hinge motions are often opening and closing motions of domains intended to expose the active site, which often would be located at the center of the motion, i.e. the hinge.

Prior work[44] shows that active sites are more likely to occur at regions of low first normal mode displacement. Such regions have been shown to coincide with hinges[26]. Here we close the loop, comparing active sites directly with the Hinge Atlas annotation and quantifying the correspondence.

In order to annotate the active site locations, we BLASTed[45] the morph sequences in the computer annotated dataset against the sequences in the Catalytic Sites Atlas and considered a morph in the hinge dataset to match a protein in the CSA if they had sequence identity ≥ 99%. This high threshold was chosen to minimize the possibility of incorrectly labeling a residue in the Hinge Atlas and thereby diminishing the significance of the results. For each such pair, we transferred the catalytic site annotation to the morph. We described earlier how to browse the CSA morphs online. Of the 214 proteins in the Hinge Atlas, 94 were annotated with active site information from the CSA; the rest had no close CSA homologs. The 94 proteins comprised the dataset for this calculation. We analyzed this set using the statistical formalism described earlier, with the following variable definitions:

*C *= distance from the nearest active site, in residues.

*c *= successively: active site residues, amino acids 1 residue away from the nearest active site residue, 2 residues away, etc.

D = 28050 residues in the dataset of 94 proteins

H = 378 hinge residues in the dataset

*d*_*c *_= residues of class *c *in the dataset

*h*_*c *_= residues of class *c *in hinges.

**Figure 2:**
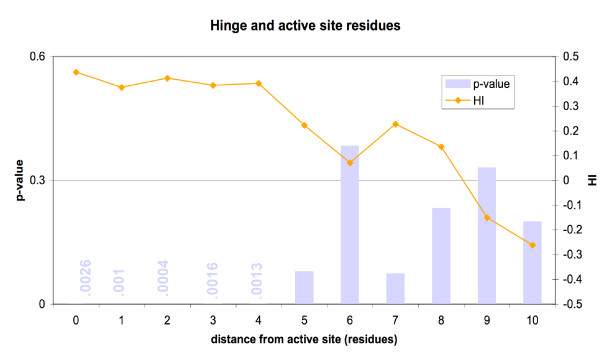
Residues within four amino acid positions of the active site are significantly more likely to be in hinges.

The results are shown in Figure 2 and Table 2. At short distances from the active sites, hinge residues were overrepresented. The active site residues and residues as much as four residues away from the nearest active site were significantly overrepresented in hinges.

**Table 2:** HI and associated p-value for hinge residue coincidence with active site, and with residues at certain distances from active site residues.

***m = distance from nearest active site (residues)***	***residues at positions m***	***hinge residues at positions m***	***Hl***_***actlvesite***_	***P-value***
0	298	11	0.44	0.0026
1	531	17	0.38	0.0010
2	487	17	0.41	0.0004
3	460	15	0.38	0.0016
4	451	15	0.39	0.0013
5	444	10	0.22	0.08
6	441	7	0.07	0.38
7	439	10	0.23	0.08
8	434	8	0.14	0.23
9	419	4	-0.15	0.33
10	406	3	-0.26	0.20

### Are hinges segregated by secondary structure?

It is generally accepted that hinges tend to avoid secondary structure. However this belief has, to our knowledge, never been tested on a quantitative basis, and indeed numerous counterexamples can be found. For instance, the hinge in calmodulin[13] and troponin C[26,46] occurs in an α-helix, and in glutamine binding protein it occurs in two parallel beta strands[26]. Thus we do not know which particular types of secondary structure are avoided or preferred, or to what degree. To obtain this information, we tabulated the number of hinge residues occurring in the various types of secondary structural elements, and compared this with the distribution of all residues, proceeding as follows.

STRIDE[47] recognizes secondary structural elements from atomic coordinates. We used this program to assign secondary structural classes to all residues in the Hinge Atlas. We then tabulated the number of residues assigned to each class, both in hinges and elsewhere in the dataset. Lastly, we calculated the *HI *scores and the p-values as before, letting *C *= secondary structural element type and *c *designate e.g. helix, coil, etc.

**Figure 3:**
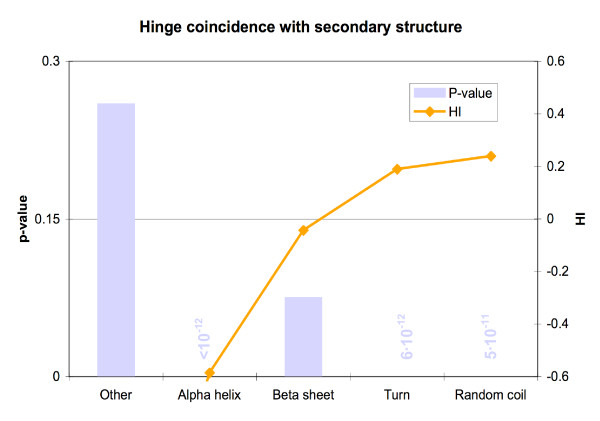
Residues in alpha helices were less likely to occur in hinges, with very high significance. Turn and coil residues, on the other hand, were more likely to be in hinges, also with high statistical significance.

We found that three types of secondary structure were differentially represented in hinges with extremely high significance. We conclude that hinges are less likely to occur in α-helices, and are more likely to occur in turns or random coils (Figure 3 and Table 3). For the user's convenience, secondary structure assignments for individual morphs can be obtained in the 'Hinge analysis tools' box on the MolMovDB morph page[9] mentioned earlier.

**Table 3:** Hinge frequency of occurrence in various types of secondary structure.

Secondary structure	Hinge Residues (count)	All residues (count)	Hinge Residues (expected)	HI_secondarystructure_	P-value
α-helix	75	18210	290	-0.587	1·8^-67^
3–10 helix	27	1937	31	-0.0577	0.27
π-helix	0	5	0		0.92
Extended					
conformation	160	11138	177	-0.0446	0.076
Isolated bridge	19	670	11	0.251	0.12
Turn	306	12472	199	0.188	5.9·10^-17^
Coil (none of the others)	286	10408	166	0.237	1.2·10^-22^
Total	873	54840			

### Are certain physicochemical properties preferred in hinge residues?

**Figure 4:**
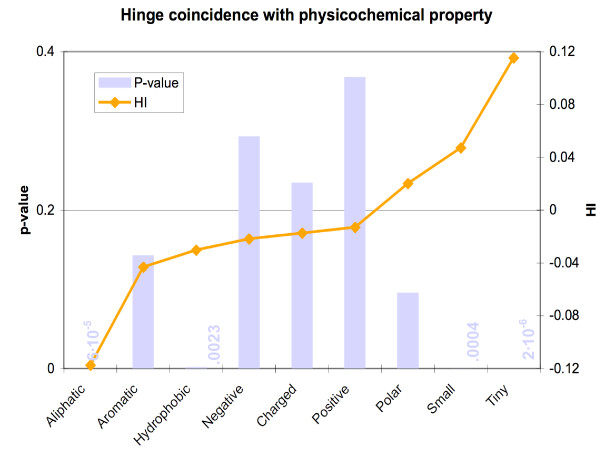
Size, aliphaticity, and hydrophobicity appear to account for much of the segregation of residues along physicochemical lines. In particular, the individually underrepresented residues (Gly, Ser, Ala) are classified as "tiny." Other underrepresented residues types (Leu, Val) are aliphatic, while still others (Phe, and again Val) are hydrophobic.

It is intuitive that certain physicochemical classes of residues (such as small and hydrophilic) would occur more frequently in hinges, and this would help explain the amino acid propensities reported earlier in this work. To check and quantify this, we grouped amino acids into several non-exlusive categories[48]. Following again our statistical treatment, we calculated *HI *scores and p-values, letting *C *= physicochemical grouping, and *c *= aliphatic, polar, charged, etc. We discovered that aliphatic and hydrophobic residues were very significantly underrepresented. Overrepresented were small and tiny residues (Figure 4 and Table 4).

**Table 4:** Hinge frequency of occurrence in various physicochemical classifications

Category	Amino acids	Hinge Residues (count)	All residues (count)	Hinge Residues (expected)	HI	P-value
Aliphatic	I, L, V	144	11915	190	-0.120	5.9·10^-05^

Aromatic	H, F, W, Y	82	5718	91	-0.0454	0.14
	A, C, G, H, I, L, K,					

Hydrophobic	M, F, T, W, Y, V	521	35278	562	-0.0326	0.0023

Negative	D, E	102	6774	108	-0.0242	0.29

Charged	R, D, E, H, Y	212	13931	227	-0.0196	0.24

Positive	R, H, Y	110	7157	114	-0.0153	0.37
	R, N, D, E, Q, H, Y,					

Polar	S, T, W, Y	488	29427	468	0.0178	0.096
	A, N, D, C, G, P, S,					

Small	T, V	502	28430	453	0.0450	0.00041

Tiny	G, A, S	253	12247	195	0.113	0.0000023

### Are hinge residues conserved in evolution?

We next investigated whether hinge residues are conserved. Since certain residue classes are preferred in hinges, one might suspect that hinge residues would be conserved. First, we BLASTed[45] each of the Hinge Atlas sequences against nrdb90, a non-redundant sequence database in which protein sequences have no more than 90% sequence identity with each other [37] Next we extracted up to 50 top-aligned sequences to a given morph to generate a multiple sequence alignment using Clustal W[49]. For each position in the multiple sequence alignment, we used the formalism developed by Schneider et al[50] to compute the information content associated with a column in the multiple sequence alignment at this position[50,51].

We sorted the residues in Hinge Atlas morphs according to the magnitude of the information content scores. We then divided the residues into five bins of equal size. If hinge residues are conserved, then there should be an enrichment of hinge residues in the top bins, which correspond to the most conserved residues. On the other hand, if hinge residues are hypermutable, there should be more of them in the bottom bins, corresponding to the least conserved residues. Because it is widely agreed that active sites should be conserved, we used the conservation of active sites as a control.

To quantify the enrichment, we calculated the HI scores as described previously. Here, *c *is a label applied to residues that ranked in a given percentile bin, e.g. the top 20% most conserved. For that bin *p*(*a*_*c*_|*h*) = *h*_*c*_/*H *is thus the ratio of the number of hinge residues in the bin divided by the total number of hinge residues. Similarly, *p*(*a*_*c*_) = *d*_*c*_/*D *is the ratio of the number of residues in the dataset in the bin divided by the grand total of residues in the dataset. To determine the statistical significance of HI scores, we calculated the p-values using the hypergeometric distribution with the *d*_*c*_, *h*_*c*_, *D*, *H *defined above.

For the control set, we performed the same calculation but made the following changes to the variable definitions:

1. Our dataset was no longer the Hinge Atlas, but rather the "Catalytic Sites Atlas (nonredundant)" set described earlier. *D *is the total number of residues in this set.

2. *a*_*c *_still represents residues in the dataset belonging to a given conservation rank bin. *d*_*c *_is the total number of residues in that bin.

3. *h*_*c *_now represents the number of *active site *residues in a given bin corresponding to *c*. Similarly, *H *represents the total number of active site residues in the dataset.

**Figure 5:**
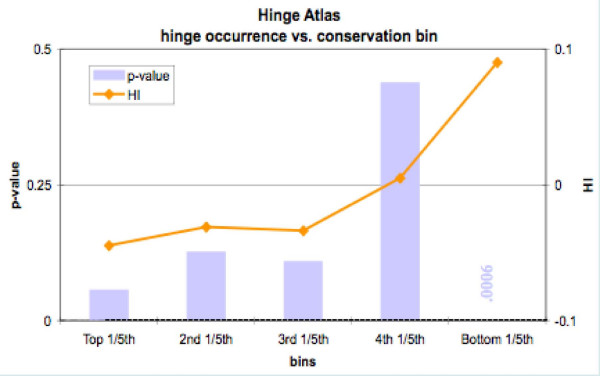
The least conserved 20% of residues are significantly more likely to appear in hinges.

We found that hinge residues distribute evenly in the top 80%, and have a slight but statistically very significant enrichment in the bottom 20% bin (Figure 5 and Table 5). Thus hinge residues are hypermutable. We observe a highly significant enrichment of active site residues in the top 20% bin, as expected. Among the 947 active site residues, 813 of them (86%) are in the highest bin, and the numbers progressively decrease in lower bins.

**Table 5:** Hinge frequency of occurrence vs. conservation bin.

	Active site residue propensity			Hinge residue propensity				
	
	Enzymes in Hinge Atlas			Hinge Atlas			Enzymes in Hinge Atlas	
	
Conservation score bin	Active site residues	HI	p-value	Hinge residues	HI	p-value	Hl	p-value
Top 1/5th	813	0.63	<10^-29^	157	-0.045	0.057	-.0093	.45
2nd 1/5th	53	-0.55	<10^-29^	162	-0.031	0.13	-.027	.30
3rd 1/5th	44	-0.63	<10^-29^	161	-0.034	0.11	-.015	.40
4th 1/5th	22	-0.94	<10^-29^	176	0.0050	0.44	-.086	.043
Bottom 1/5th	15	-1.1	<10^-29^	213	0.090	0.00061	.113	.0029
	947			869				

The Hinge Atlas pools enzymes together with non-catalytic proteins. We reasoned therefore that perhaps only hinges in non-catalytic proteins are hypermutable, and that if we analyzed a set consisting only of enzymes, then the propensity of active sites to occur in hinges would lead to conservation, rather than hypermutability of hinge residues for that set.

**Figure 6:**
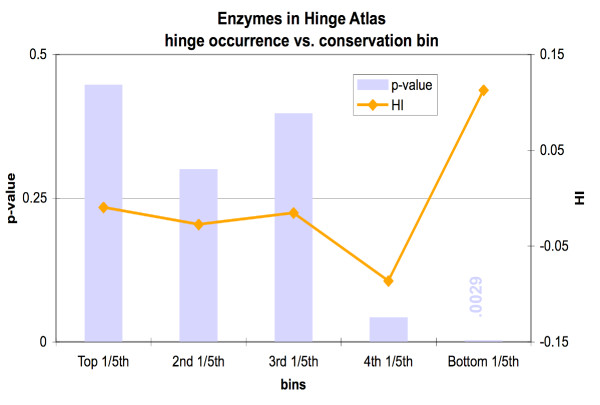
Since active sites residues are enriched in hinges, we performed a separate conservation check on hinge residues in the 94 Hinge Atlas proteins with CSA annotation. We found that even in this set, the least conserved 1/5^th ^of amino acids in each protein tended to contain significantly more hinge residues. The fourth bin was sparse in hinge residues, but at a p-value of 0.043, the significance of this was marginal.

To test this idea, we decided to calculate the propensity of hinges to occur in specific bins of conservation score, for the 94 proteins in the Hinge Atlas with CSA annotation, rather than for the larger set of 214. For this set we also found that hinge residues occur more frequently among the 20% least conserved residues for each protein (Figure 6, Table 5). At a p-value of 0.003, the confidence in this result is high.

Even this test, however pools together hinges that are near the active site (or contain one or more active site residues) with hinges that occur at some distance from it. So we selected from the 94 proteins a small set that had at least one active site residue in the hinge, and removed the active site residues themselves. We then calculated the propensity of hinge residues to occur in the five conservation bins. This set was found to be too small, however, and statistical significance was too low to draw a conclusion (data not shown). A study using the set of fragment hinge motions described earlier was similarly inconclusive.

The hypermutability of hinge residues that we found is reasonable because hinge residues tend to be on the surface of proteins (see below) rather than in the more highly conserved core. Hinges are less likely to be buried inside domains because they would then be highly coordinated with near neighbors and hence less flexible. The apparent contradiction of hypermutability on the one hand and enrichment of active sites on the other is dealt with in the Discussion section.

### Are hinge residues more likely to occur on the surface?

**Figure 7:**
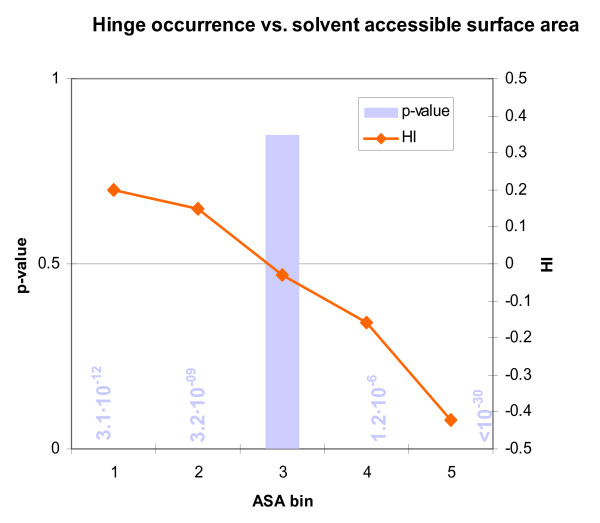
Hinge residues tend to be on the surface, since steric clashes would often prevent them from being in the core. We computed the solvent accessible surface area for the backbone atoms of all residues in the Hinge Atlas and binned the residues by this quantity. Bin #1 contains the 20% of all residues with the largest solvent accessible surface area, and bin #5 contains the 20% of residues with the smallest solvent accessible surface area. The first two bins (together representing the 40% of residues with highest surface area) are enriched with hinges in a highly significant manner. Conversely, the last two bins (lowest 40% ASA) are significantly low in hinges.

To support our argument that hinge residues are hypermutable partly because they occur on the surface, we quantified the degree to which the latter is the case. To do this, we used a solvent accessible surface area (ASA) calculation program [52,53] with a probe radius of 1.4Å. The ASA of each of the backbone heavy atoms (amide nitrogen, α-carbon, carbonyl carbon and oxygen) was calculated and summed for each residue in each protein in the Hinge Atlas. We then binned the residues by this quantity. Lastly, we counted the number of hinge residues in each bin and calculated HI and p-value as before. As expected, bin #1 (containing the 20% of residues with highest ASA) was significantly enriched with hinges (Figure 7; Table 6). Bin #2 was also highly enriched, while bins #4 and #5 had fewer hinges, all with extremely high significance.

**Table 6:** Hinge Index and p-value for differential representation of residues binned by solvent accessible surface area (bin #1 represents largest area).

***Bin***	***Hinge residues***	***HI***	***p-value***
1	277	0.200	1.1·10^-16^
2	246	0.149	3.3·10^-09^
3	163	-0.030	0.849
4	121	-0.159	1.2·10^-06^
5	66	-0.422	6.6·10^-25^
	873		

Bins 1–4 contain 10968 total residues and bin 5 contains 10967.

### How many hinge sites appear in each protein?

Perhaps the simplest hinge consists of a single point on the chain separating two rigid regions. However it is also possible for the chain to pass multiple times through the same region, or to have multiple independent hinge regions. This leads to the question, how many proteins had single hinge points, versus a larger number of hinge points? We answer this question in Table 7. Most morphs had three or fewer hinge points.

**Table 7:** Number of hinge points per protein in the Hinge Atlas

Number of hinge points	Number of protein pairs (morphs)
1	76
2	75
3	56
4	6
5	1
Total:	214

### Can hinges be predicted by a simple GOR-like method?

GOR[30,31] method is useful for predicting secondary structure from sequence with fair accuracy. We implemented a GOR-like method to determine whether sequence contained enough information for hinge prediction. We divided the dataset into a training set and a test set for this study. The log-odds frequency of occurrence of amino acids in the training set were tabulated not only at a given hinge residue, but also at positions ranging from -8 to +8 from the given residue in sequence space. For simplicity, hinge residues at positions less than eight residues from either end of the chain were not included.

Once the table was generated, it was used on the test set. The score for a given residue was taken to be the sum of the scores for the residues in positions -8 to +8 from that residue. The scores were computed for all residues in the test set, except those less than eight residues from either end of the chain. The idea is that a threshold score can be chosen and residues scoring higher than this threshold are considered more likely to be hinges. Note that where Robson and Suzuki used a different fitting parameter for each type of secondary structure, we used no fitting parameter, since we were interested in only one "secondary structure": the hinges. The rates of true and false positives and negatives were calculated for each choice of score threshold over a range.

Our training set numbered 136 proteins from the computer annotated set. We tested the method on a test set of 137 proteins from the same set and obtained a ROC curve[35] (not shown; ROC curves are explained later in this work). The area under this curve was nearly 0.5, indicating negligible predictive value.

### Hinge prediction by combining sequence features

As the GOR-like method did not work well, we sought to measure the predictive power of the various sequence features studied above. The HI scores we have reported provide an intuitive means of weighing the relative predictive value of each sequence feature. We show how to combine the HI scores for several features in order to make a more powerful predictor, which we call HingeSeq. We define this predictor as follows:

HS(i)=log⁡10(p(aj|h)p(ak|h)p(al|h)p(aj)p(ak)p(al))=HIamino⋅acid(i)+HIsecondary⋅structure(i)+HIactive⋅site(i)
 MathType@MTEF@5@5@+=feaafiart1ev1aaatCvAUfKttLearuWrP9MDH5MBPbIqV92AaeXatLxBI9gBaebbnrfifHhDYfgasaacH8akY=wiFfYdH8Gipec8Eeeu0xXdbba9frFj0=OqFfea0dXdd9vqai=hGuQ8kuc9pgc9s8qqaq=dirpe0xb9q8qiLsFr0=vr0=vr0dc8meaabaqaciaacaGaaeqabaqabeGadaaakeaacqWGibascqWGtbWucqGGOaakcqWGPbqAcqGGPaqkcqGH9aqpcyGGSbaBcqGGVbWBcqGGNbWzdaWgaaWcbaGaeGymaeJaeGimaadabeaakmaabmaabaWaaSaaaeaacqWGWbaCcqGGOaakcqWGHbqydaWgaaWcbaGaemOAaOgabeaakiabcYha8jabdIgaOjabcMcaPiabdchaWjabcIcaOiabdggaHnaaBaaaleaacqWGRbWAaeqaaOGaeiiFaWNaemiAaGMaeiykaKIaemiCaaNaeiikaGIaemyyae2aaSbaaSqaaiabdYgaSbqabaGccqGG8baFcqWGObaAcqGGPaqkaeaacqWGWbaCcqGGOaakcqWGHbqydaWgaaWcbaGaemOAaOgabeaakiabcMcaPiabdchaWjabcIcaOiabdggaHnaaBaaaleaacqWGRbWAaeqaaOGaeiykaKIaemiCaaNaeiikaGIaemyyae2aaSbaaSqaaiabdYgaSbqabaGccqGGPaqkaaaacaGLOaGaayzkaaGaeyypa0JaemisaGKaemysaK0aaSbaaSqaaGqaciab=fgaHjab=1gaTjab=LgaPjab=5gaUjab=9gaVjabgwSixlabdggaHjabdogaJjabdMgaPjabdsgaKbqabaGccqGGOaakcqWGPbqAcqGGPaqkcqGHRaWkcqWGibascqWGjbqsdaWgaaWcbaGae83CamNae8xzauMae83yamMae83Ba8Mae8NBa4Mae8hzaqMae8xyaeMae8NCaiNae8xEaKNaeyyXICTaem4CamNaemiDaqNaemOCaiNaemyDauNaem4yamMaemiDaqNaemyDauNaemOCaiNaemyzaugabeaakiabcIcaOiabdMgaPjabcMcaPiabgUcaRiabdIeaijabdMeajnaaBaaaleaacqWGHbqycqWGJbWycqWG0baDcqWGPbqAcqWG2bGDcqWGLbqzcqGHflY1cqWGZbWCcqWGPbqAcqWG0baDcqWGLbqzaeqaaOGaeiikaGIaemyAaKMaeiykaKcaaa@B40A@

Equation 4

For simplicity, statistical independence of the various features was assumed in creating this definition. Here the *i*'s correspond to individual amino acids in the protein sequence. For each *i*, *j *designates one of the 20 amino acid types, *k *designates the secondary structural classification, and *l *designates active site versus non-active site classification.

Thus *HI*_*amino*·*acid*_(*i*) is assigned according to residue type by looking up the corresponding value in Table 1. Similarly, *HI*_*secondary*·*structure*_(*i*) isobtained according to secondary structure type from Table 3. Following Table 2 approximately, we assign *HI*_*active*·*site*_(*i*) as 0.4 for residues four or fewer amino acid positions away from the nearest active site residue, and 0.0 elsewhere. The highest values of *HS*(*i*) correspond to residues most likely to occur in hinges.

Clearly, extending this method is only a matter of obtaining amino acid propensities to occur in hinges according to additional classifications. The resulting index can then simply be included as an additional term in the above formula, with no need for adjustable weighting factors.

We evaluated the statistical significance of this measure much as for the individual sequence features. We counted the number of residues in the Hinge Atlas with a HingeSeq score above 0.5, and within that set the number of hinge residues. We compared this to the total number of hinges and the population size of the Hinge Atlas (Table 8). Using the cumulative hypergeometric distribution as before, we computed a p-value of order 10^-12^, thus the measure shows high statistical significance. However since only about 5% of the residues scoring over 0.5 were annotated hinges, HingeSeq is not likely to be sensitive enough to be used alone for hinge prediction.

**Table 8:** Statistical analysis of HingeSeq predictor.

Total resid. in Hinge Atlas	54839
Hinges in Hinge Atlas	873
Total residues with HingeSeq score > .5	924
Hinge residues with HingeSeq score > .5	48
p-value	1.5·10^-12^

The low p-value indicates that the predictor results have high statistical significance. However the low sensitivity limits its potential predictive value.

We nonetheless wished to show that HingeSeq is predictive, rather simply reflectling peculiarities of the dataset. To this end, we divided the 214 proteins of the Hinge Atlas into a training set numbering 161 proteins, and a test set numbering 53. Of the 214 Hinge Atlas proteins, the 94 proteins with annotation from the CSA were apportioned such that 71 were included in the training set and 23 in the test set. We tested the performance of the predictor by means of ROC (Receiver Operating Characteristic) curves. We need to define a few terms in order to use these:

Test positives: Residues with *HS*(*i*)greater than or equal to a certain threshold.

Test negatives: Residues with *HS*(*i*)less than a certain threshold.

Gold standard positives: Residues annotated as hinges in the Hinge Atlas.

Gold standard negatives: Residues which are not in hinges according to the Hinge Atlas annotation.

True positives (TP): Those residues that are both test positives and gold standard positives.

True negatives (TN): Residues that are both test negatives and gold standard negatives.

False positives (FP): Residues that are test positives and gold standard negatives.

False negatives (FN): Residues that are test negatives and gold standard positives.

sensitivity=TPTP+FN
 MathType@MTEF@5@5@+=feaafiart1ev1aaatCvAUfKttLearuWrP9MDH5MBPbIqV92AaeXatLxBI9gBaebbnrfifHhDYfgasaacH8akY=wiFfYdH8Gipec8Eeeu0xXdbba9frFj0=OqFfea0dXdd9vqai=hGuQ8kuc9pgc9s8qqaq=dirpe0xb9q8qiLsFr0=vr0=vr0dc8meaabaqaciaacaGaaeqabaqabeGadaaakeaacqWGZbWCcqWGLbqzcqWGUbGBcqWGZbWCcqWGPbqAcqWG0baDcqWGPbqAcqWG2bGDcqWGPbqAcqWG0baDcqWG5bqEcqGH9aqpdaWcaaqaaiabdsfaujabdcfaqbqaaiabdsfaujabdcfaqjabgUcaRiabdAeagjabd6eaobaaaaa@450B@

specificity=TNFP+TN
 MathType@MTEF@5@5@+=feaafiart1ev1aaatCvAUfKttLearuWrP9MDH5MBPbIqV92AaeXatLxBI9gBaebbnrfifHhDYfgasaacH8akY=wiFfYdH8Gipec8Eeeu0xXdbba9frFj0=OqFfea0dXdd9vqai=hGuQ8kuc9pgc9s8qqaq=dirpe0xb9q8qiLsFr0=vr0=vr0dc8meaabaqaciaacaGaaeqabaqabeGadaaakeaacqWGZbWCcqWGWbaCcqWGLbqzcqWGJbWycqWGPbqAcqWGMbGzcqWGPbqAcqWGJbWycqWGPbqAcqWG0baDcqWG5bqEcqGH9aqpdaWcaaqaaiabdsfaujabd6eaobqaaiabdAeagjabdcfaqjabgUcaRiabdsfaujabd6eaobaaaaa@44A9@

1−specificity=FPFP+TN
 MathType@MTEF@5@5@+=feaafiart1ev1aaatCvAUfKttLearuWrP9MDH5MBPbIqV92AaeXatLxBI9gBaebbnrfifHhDYfgasaacH8akY=wiFfYdH8Gipec8Eeeu0xXdbba9frFj0=OqFfea0dXdd9vqai=hGuQ8kuc9pgc9s8qqaq=dirpe0xb9q8qiLsFr0=vr0=vr0dc8meaabaqaciaacaGaaeqabaqabeGadaaakeaacqaIXaqmcqGHsislcqWGZbWCcqWGWbaCcqWGLbqzcqWGJbWycqWGPbqAcqWGMbGzcqWGPbqAcqWGJbWycqWGPbqAcqWG0baDcqWG5bqEcqGH9aqpdaWcaaqaaiabdAeagjabdcfaqbqaaiabdAeagjabdcfaqjabgUcaRiabdsfaujabd6eaobaaaaa@466E@

**Figure 8:**
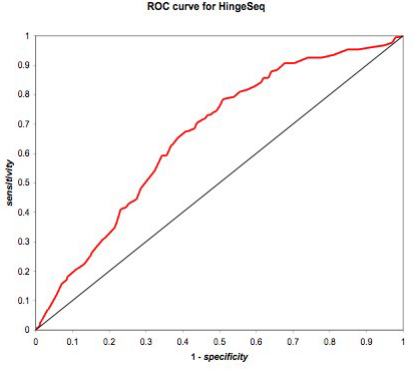
The thick red trace represents HingeSeq performance against the Hinge Atlas annotation in the test set of 53 proteins. The diagonal black line represents the performance of a completely random predictor, with area under the curve of 0.5. HingeSeq is seen to have substantial predictive power, since it encloses significantly greater area.

The ROC curve is simply a plot of the true positive rate (same as sensitivity) vs. false positive rate (1-specificity), for each value of the threshold, as the threshold is varied from +1 to -1, a range which included all possible values of *HS*(*i*). For a good predictor, the true positive rate will increase faster than the false positive rate as the threshold is lowered, and the area under the curve will be significantly greater than 0.5. The ROC curve is shown in Figure 8. Although work remains to be done before sequence-based hinge prediction can be relied upon exclusively, HingeSeq displays significant ability to detect potential for flexibility.

## Checking for dataset bias

These findings assume that the dataset used does not contain significant bias or artifacts, either in the composition of the entire dataset or of the hinges within it. To substantiate this, we performed various studies as follows.

### Bias in amino acid composition and functional classification

**Figure 9:**
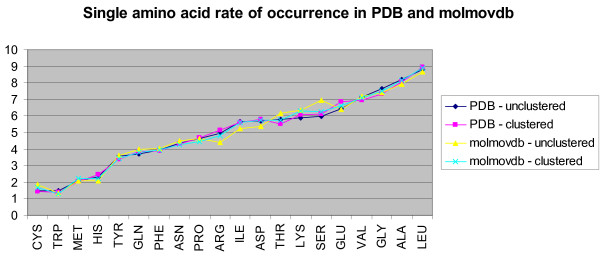
To check for possible database bias, we computed the amino acid composition of MolMovDB and found that it follows that of the PDB, from which it is largely compiled.

In order to find out whether the MolMovDB database contained any bias in amino acid composition, we extracted the sequences of all the morphs in MolMovDB and counted the total occurrence of each residue type. Suspecting that redundancies might bias the result, we clustered the sequences and recounted the amino acid residues in the same way. We compared these numbers to publicly available amino acid frequencies of occurrence for the PDB (Protein Data Bank)[54] (Figure 9). The amino acid frequency of occurrence for the clustered MolMovDB morphs was found to be essentially that of the PDB, from which it was created, therefore no particular database bias is in evidence.

We also sought to determine whether there existed a bias towards particular protein classes, in either the Hinge Atlas or the nonredundant set of MolMovDB morphs from which it was compiled. To do this, we first counted the number of times each top-level Gene Ontology (GO) term under the "molecular function" ontology was associated with a protein in the Hinge Atlas. Where the annotation was given for deeper levels, we traced up the hierarchical tree to retrieve the corresponding top level term in the ontology. Thus we found, for example, that 14 proteins in the Hinge Atlas were associated with the term "nucleic acid binding." We repeated this procedure for the PDB as a whole as well as for the non-redundant set of 1508 morphs in MolMovDB from which the Hinge Atlas was compiled. The results for the 10 most frequently encountered GO terms are shown in Table 9.

**Table 9:** Frequency of Gene Ontology terms in PDB vs. Hinge Atlas

Counts in PDB	Counts in Hinge Atlas	Gene Ontology term
7110	32	hydrolase activity
3862	16	transferase activity
3721	14	nucleic acid binding
3629	23	ion binding
2848	15	nucleotide binding oxidoreductase
2693	17	activity
1748	6	molecular_function
1553	6	protein binding electron transporter
1088	4	activity
929	5	lyase activity
...	...	etc

To compare the Hinge Atlas counts to the PDB counts in an overall fashion, we used the chi-square distribution with 162 degrees of freedom (from 163 GO terms and 2 datasets) and obtained a chi-square value of 121.1. This corresponds to a p-value of 0.9931, so there is no statistically significant difference in the distribution of these terms in the Hinge Atlas vs. the entire Protein Data Bank.

### Statistical comparison of datasets

The Hinge Atlas and computer annotated sets were compiled differently, therefore one might suspect that the hinges from one set might comprise a statistically different population from the hinges of the other set. If this were the case, then one of the two sets would be preferable to the other, otherwise if the populations were essentially the same then the two sets could potentially be used interchangeably. It is therefore necessary to quantitatively compare these two populations. It is also necessary to confirm that within one set, the hinge residues are a statistically distinct population from the rest of the set; if this were not true then the amino acid propensity data reported earlier would not be meaningful.

**Figure 10:**
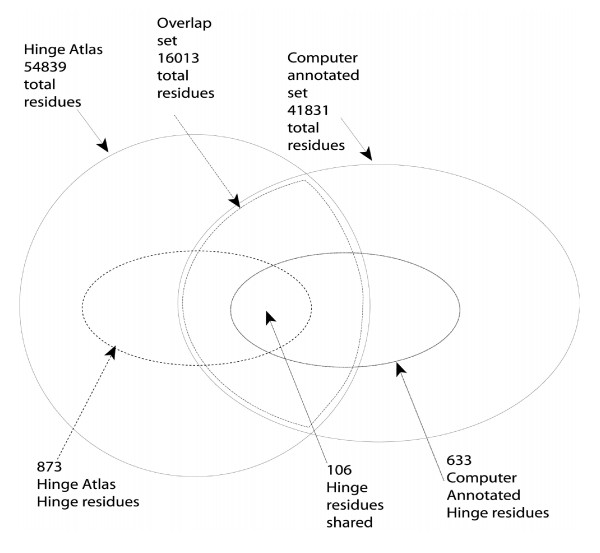
Although the Hinge Atlas and computer annotated set have a significant overlap, they are statistically different sets. Importantly, the hinge residues within these sets are different from each other, despite sharing 106 residues.

Although the Hinge Atlas and the computer annotated set share a total of 16013 residues, only 106 (~0.7%) (Figure 10) of these are hinge residues. This is another reason to suspect that the hinge population of the Hinge Atlas is statistically different from the hinge population of the computer annotated set. To test this, we computed the chi-square value for Hinge Atlas hinges vs. computer annotated hinges, and obtained a p-value of 0.03. Therefore, the Hinge Atlas hinges are different from the computer annotated hinges. The chi-square value describing the difference between amino acid frequency of occurrence in the hinge vs. non-hinge subsets of the Hinge Atlas was 99.01. With 19 degrees of freedom (from 20 amino acids and 2 sets) this corresponds to a p-value below 10^-4 ^(Table 10). Therefore, the hinge residues are shown with high confidence to be different from non-hinge residues in the Hinge Atlas. A similar calculation yielded a p-value of 0.017 for the computer annotated set. Therefore we conclude that the hinge and non-hinge populations are different for the computer annotated set, as well.

**Table 10:** The hinges within the computer annotated set comprise a distinct population from the rest of the set (p-value = 0.017).

	**Computer annotated set Hinge vs. non-hinge residues**	**Hinge Atlas Hinge vs. non-hinge residues**	**Hinge Atlas hinges vs. Computer annotated hinges**
*Chi-square*	34.37	99.01	31.84
*DOFs*	19	19	19
*p-value*	0.017	<10^-4^	0.03

The same is true for the Hinge Atlas set.

Hinges in the Hinge Atlas are likely to be a distinct population from hinges in the computer annotated set (p-value 0.03); therefore for future studies one or the other should be used, and we recommend that it be the Hinge Atlas.

We conclude from this calculation for both the Hinge Atlas and computer annotated set, the hinge population is different from the non-hinge population, therefore statistically significant information can be extracted from both. However the hinge population of the Hinge Atlas is different from that of the computer annotated set, albeit with much lower significance. We argue that one of the two sets should therefore be preferred for statistical studies. The preferred set should be the Hinge Atlas since the computer annotated set contains numerous annotations which are slightly different from the correct and visually verifiable hinge location.

### Similarity within morph pairs

We next asked the question, do the morphs in the Hinge Atlas reflect intrinsic flexibility of the protein, or is the apparent conformational change the result of sequence differences between the two structures in the pair? That is, do the morphs display motions observable in a single protein, or do they instead represent evolutionary change? To answer this we counted the number of times both structures in the morph came from the same vs different organisms. Of the 214 morphs, 123 had structures downloaded directly from the PDB rather than uploaded by users, and also had valid source organism data. For 109 of the 123, both proteins in the pair came from the same species, while for 14 the two proteins came from different species. Of the 14, 11 pairs were of proteins that were somewhat related to each other (7 pairs of bacterial, and 4 pairs of mammalian), while only three pairs were comprised of two proteins from different kingdoms. Thus the conformational changes are likely to reflect experimentally observable motions rather than evolutionary effects.

### Resampling of hinge residues

As a further test of confidence in the Hinge Atlas, we decided to look for sampling artifacts in the hinge set. Resampling[32] or bootstrapping[55] is a technique suited for this purpose. We bootstrapped the *frequency *of occurrence of amino acid types. The method consists of drawing random samples and computing the frequency of occurrence of a given amino acid type in that sample. We present the results for glycine, the residue type most overrepresented in hinges.

We randomly chose 1/8 of the 214 proteins in the Hinge Atlas. The sample was labelled with an index *j*. Within that sample we counted the following:

Hj*
 MathType@MTEF@5@5@+=feaafiart1ev1aaatCvAUfKttLearuWrP9MDH5MBPbIqV92AaeXatLxBI9gBaebbnrfifHhDYfgasaacH8akY=wiFfYdH8Gipec8Eeeu0xXdbba9frFj0=OqFfea0dXdd9vqai=hGuQ8kuc9pgc9s8qqaq=dirpe0xb9q8qiLsFr0=vr0=vr0dc8meaabaqaciaacaGaaeqabaqabeGadaaakeaacqWGibasdaqhaaWcbaGaemOAaOgabaGamaiVcQcaQaaaaaa@316F@ : the number of hinge residues of all amino acid types in sample *j*,

H˜j*
 MathType@MTEF@5@5@+=feaafiart1ev1aaatCvAUfKttLearuWrP9MDH5MBPbIqV92AaeXatLxBI9gBaebbnrfifHhDYfgasaacH8akY=wiFfYdH8Gipec8Eeeu0xXdbba9frFj0=OqFfea0dXdd9vqai=hGuQ8kuc9pgc9s8qqaq=dirpe0xb9q8qiLsFr0=vr0=vr0dc8meaabaqaciaacaGaaeqabaqabeGadaaakeaacuWGibasgaacamaaDaaaleaacqWGQbGAaeaacWaG8kOkaOcaaaaa@317E@ : the number of NON-hinge residues of all amino acid types in sample *j*,

hj*
 MathType@MTEF@5@5@+=feaafiart1ev1aaatCvAUfKttLearuWrP9MDH5MBPbIqV92AaeXatLxBI9gBaebbnrfifHhDYfgasaacH8akY=wiFfYdH8Gipec8Eeeu0xXdbba9frFj0=OqFfea0dXdd9vqai=hGuQ8kuc9pgc9s8qqaq=dirpe0xb9q8qiLsFr0=vr0=vr0dc8meaabaqaciaacaGaaeqabaqabeGadaaakeaacqWGObaAdaqhaaWcbaGaemOAaOgabaGamaiVcQcaQaaaaaa@31AF@ (*a*_*GLY*_) : the number of glycine residues in hinges in sample *j*,

h˜j*
 MathType@MTEF@5@5@+=feaafiart1ev1aaatCvAUfKttLearuWrP9MDH5MBPbIqV92AaeXatLxBI9gBaebbnrfifHhDYfgasaacH8akY=wiFfYdH8Gipec8Eeeu0xXdbba9frFj0=OqFfea0dXdd9vqai=hGuQ8kuc9pgc9s8qqaq=dirpe0xb9q8qiLsFr0=vr0=vr0dc8meaabaqaciaacaGaaeqabaqabeGadaaakeaacuWGObaAgaacamaaDaaaleaacqWGQbGAaeaacWaG8kOkaOcaaaaa@31BE@ (*a*_*GLY*_) : the number of glycines in NON-hinge residues in sample *j*,

fj*(aGLY)=hj*(aGLY)Hj*
 MathType@MTEF@5@5@+=feaafiart1ev1aaatCvAUfKttLearuWrP9MDH5MBPbIqV92AaeXatLxBI9gBaebbnrfifHhDYfgasaacH8akY=wiFfYdH8Gipec8Eeeu0xXdbba9frFj0=OqFfea0dXdd9vqai=hGuQ8kuc9pgc9s8qqaq=dirpe0xb9q8qiLsFr0=vr0=vr0dc8meaabaqaciaacaGaaeqabaqabeGadaaakeaacqWGMbGzdaqhaaWcbaGaemOAaOgabaGamaiPcQcaQaaakiabcIcaOiabdggaHnaaBaaaleaacqWGhbWrcqWGmbatcqWGzbqwaeqaaOGaeiykaKIaeyypa0ZaaSaaaeaacqWGObaAdaqhaaWcbaGaemOAaOgabaGamaiVcQcaQaaakiabcIcaOiabdggaHnaaBaaaleaacqWGhbWrcqWGmbatcqWGzbqwaeqaaOGaeiykaKcabaGaemisaG0aa0baaSqaaiabdQgaQbqaaiadasQGQaGkaaaaaaaa@49B7@ : the *sample frequency *of occurrence of glycines within hinges within sample *j*, and

f˜j*(aGLY)=h˜j*(aGLY)H˜j*
 MathType@MTEF@5@5@+=feaafiart1ev1aaatCvAUfKttLearuWrP9MDH5MBPbIqV92AaeXatLxBI9gBaebbnrfifHhDYfgasaacH8akY=wiFfYdH8Gipec8Eeeu0xXdbba9frFj0=OqFfea0dXdd9vqai=hGuQ8kuc9pgc9s8qqaq=dirpe0xb9q8qiLsFr0=vr0=vr0dc8meaabaqaciaacaGaaeqabaqabeGadaaakeaacuWGMbGzgaacamaaDaaaleaacqWGQbGAaeaacWaGKkOkaOcaaOGaeiikaGIaemyyae2aaSbaaSqaaiabdEeahjabdYeamjabdMfazbqabaGccqGGPaqkcqGH9aqpdaWcaaqaaiqbdIgaOzaaiaWaa0baaSqaaiabdQgaQbqaaiadaYRGQaGkaaGccqGGOaakcqWGHbqydaWgaaWcbaGaem4raCKaemitaWKaemywaKfabeaakiabcMcaPaqaaiqbdIeaizaaiaWaa0baaSqaaiabdQgaQbqaaiadasQGQaGkaaaaaaaa@49E4@ : the *sample frequency *of occurrence of glycines among NON-hinge residues in sample *j*.

We repeated the above for for *j *= 1 to 10000, randomizing the sample each time. For the case of *a*_*GLY *_= glycines, we generated bins 0.02 wide and counted the number of times values of fj*
 MathType@MTEF@5@5@+=feaafiart1ev1aaatCvAUfKttLearuWrP9MDH5MBPbIqV92AaeXatLxBI9gBaebbnrfifHhDYfgasaacH8akY=wiFfYdH8Gipec8Eeeu0xXdbba9frFj0=OqFfea0dXdd9vqai=hGuQ8kuc9pgc9s8qqaq=dirpe0xb9q8qiLsFr0=vr0=vr0dc8meaabaqaciaacaGaaeqabaqabeGadaaakeaacqWGMbGzdaqhaaWcbaGaemOAaOgabaGamaiScQcaQaaaaaa@319F@(*a*_*GLY*_) and f˜j*
 MathType@MTEF@5@5@+=feaafiart1ev1aaatCvAUfKttLearuWrP9MDH5MBPbIqV92AaeXatLxBI9gBaebbnrfifHhDYfgasaacH8akY=wiFfYdH8Gipec8Eeeu0xXdbba9frFj0=OqFfea0dXdd9vqai=hGuQ8kuc9pgc9s8qqaq=dirpe0xb9q8qiLsFr0=vr0=vr0dc8meaabaqaciaacaGaaeqabaqabeGadaaakeaacuWGMbGzgaacamaaDaaaleaacqWGQbGAaeaacWaGWkOkaOcaaaaa@31AE@(*a*_*GLY*_) occurred in each interval.

The results for glycine are shown in Figure 10. If sampling artifacts were present in the dataset, these might manifest themselves as departures from the Gaussian distribution. This was not observed; the distribution is approximately normal. As a further measure of our confidence in the overrepresentation of this particular amino acid, we can obtain the significance by an alternate (conservative) test as follows.

**Figure 11:**
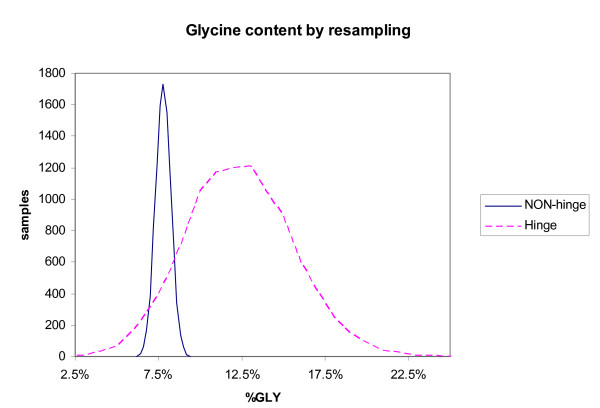
Histogram of f˜j*
 MathType@MTEF@5@5@+=feaafiart1ev1aaatCvAUfKttLearuWrP9MDH5MBPbIqV92AaeXatLxBI9gBaebbnrfifHhDYfgasaacH8akY=wiFfYdH8Gipec8Eeeu0xXdbba9frFj0=OqFfea0dXdd9vqai=hGuQ8kuc9pgc9s8qqaq=dirpe0xb9q8qiLsFr0=vr0=vr0dc8meaabaqaciaacaGaaeqabaqabeGadaaakeaacuWGMbGzgaacamaaDaaaleaacqWGQbGAaeaacWaGWkOkaOcaaaaa@31AE@(*a*_*GLY*_) (*sample frequency *of glycine among NON-hinge residues, blue trace) and fj*
 MathType@MTEF@5@5@+=feaafiart1ev1aaatCvAUfKttLearuWrP9MDH5MBPbIqV92AaeXatLxBI9gBaebbnrfifHhDYfgasaacH8akY=wiFfYdH8Gipec8Eeeu0xXdbba9frFj0=OqFfea0dXdd9vqai=hGuQ8kuc9pgc9s8qqaq=dirpe0xb9q8qiLsFr0=vr0=vr0dc8meaabaqaciaacaGaaeqabaqabeGadaaakeaacqWGMbGzdaqhaaWcbaGaemOAaOgabaGamaiScQcaQaaaaaa@319F@(*a*_*GLY*_) (*sample *frequency of glycine among hinge residues, dashed red trace). The sample frequency of glycine residues among NON-hinge residues in bins containing 1/8th of all Hinge Atlas proteins was found to average 0.078. The sample frequency of glycine among hinge residues in bins containing 1/8th of all Hinge Atlas proteins was found to average 0.124. The standard deviation was considerably larger for the hinge set, since this is a small subset of the Hinge Atlas.

Since the distribution is approximately Gaussian the standard deviation of the difference between means should be obtainable by summing the standard deviations of the two sample frequencies in quadrature[56]. The z-score of the difference is obtained by dividing the difference between the *average *sample frequencies by the the thus-obtained standard deviation:

z=f¯GLY*−f˜¯GLY*(σGLY)2+(σ˜GLY)2=1.42
 MathType@MTEF@5@5@+=feaafiart1ev1aaatCvAUfKttLearuWrP9MDH5MBPbIqV92AaeXatLxBI9gBaebbnrfifHhDYfgasaacH8akY=wiFfYdH8Gipec8Eeeu0xXdbba9frFj0=OqFfea0dXdd9vqai=hGuQ8kuc9pgc9s8qqaq=dirpe0xb9q8qiLsFr0=vr0=vr0dc8meaabaqaciaacaGaaeqabaqabeGadaaakeaacqWG6bGEcqGH9aqpdaWcaaqaaiqbdAgaMzaaraWaa0baaSqaaiabdEeahjabdYeamjabdMfazbqaaiadasQGQaGkaaGccqGHsislcuWGMbGzgaacgaqeamaaDaaaleaacqWGhbWrcqWGmbatcqWGzbqwaeaacWaGKkOkaOcaaaGcbaWaaOaaaeaacqGGOaakiiGacqWFdpWCdaWgaaWcbaGaem4raCKaemitaWKaemywaKfabeaakiabcMcaPmaaCaaaleqabaGaeGOmaidaaOGaey4kaSIaeiikaGIaf83WdmNbaGaadaWgaaWcbaGaem4raCKaemitaWKaemywaKfabeaakiabcMcaPmaaCaaaleqabaGaeGOmaidaaaqabaaaaOGaeyypa0JaeGymaeJaeiOla4IaeGinaqJaeGOmaidaaa@54CB@

From the cumulative Gaussian distribution[57], events 1.42 or more standard deviations from the mean have a probability of occurrence of 0.077, giving us an additional measure of confidence that the distribution of glycines is different in the hinge vs. non-hinge sets. Note that we would expect this to be a conservative estimate of the significance (p-value is actually much lower, since the process of resampling subdivides the dataset). The main point of this analysis is that the sample is not biased by particular anomalous proteins.

## Discussion

Correlations were found between hinges and several sequence features. We found that some amino acid types are overrepresented in hinges, and much of this can be explained on the basis of physicochemical properties. Small residues appear to be preferred, especially the "tiny Ser, Gly, and Ala. Aliphatic and hydrophobic residues tend not to be in hinges. We found that residues within four amino acid positions of an active site are significantly more likely to be hinges. This is most likely related to the fact that hinge bending motion is often related to the catalytic mechanism of the enzyme. Active site residues most logically occur inside the binding cleft and therefore are likely to be in the hinge or close by. Some of these results are intuitive, but are nonetheless useful in buttressing the less expected results. Further, even the intuitive results have in many cases never been rigorously tested or put on a quantitative footing.

Surprisingly, hypermutable residues are more likely than conserved residues to occur in hinges. This was found to be true not only for the Hinge Atlas set of 214 proteins (which includes proteins with no annotated active sites), but also for the subset of 94 enzymes with CSA annotation (Figure 5, Figure 6). This may appear to contradict our earlier result that active site residues and their near neighbors are enriched in hinges. However although the catalytic residue enrichment has very high statistical significance, the number of active site residues in hinges is still small compared to the total number of residues in hinges. Thus their presence is insufficient to counter the wider tendency of hinge residues to be hypermutable. Also, the near neighbors of active site residues have no particular reason to be conserved and thus their enrichment in hinges seems unlikely to counter the tendency toward hypermutability.

This raises the question, why would residues that are functionally important not be conserved? The answer may be that it is the intricate network of interactions within the hydrophobic core of rigid regions on either side of the hinge that needs to be conserved[58], and not the hinges themselves. The importance of the stability of these domains rather than of any detailed properties of the hinges themselves is underscored by the significant success of *structure*-based hinge predictors which analyze the interactions within the domains and between the domains and the solvent, but which pay no particular attention to the hinge region itself (Flores and Gerstein, submitted), or which implicitly[59] or explicitly[60] find highly interconnected regions of the protein.

One might also ask, is it possible that co-evolution (alternatively called compensatory mutation or mutational correlation) occurs in hinge residues even in the absence of independent (single-site) conservation? Repeatedly investigators have found that co-evolving residue pairs tend to be proximal in space[61] and *stabilize *proteins, for instance by periodically bridging consecutive turns of α-helices or by interacting across the contact interface between two such helices[62]. This is an active area of research with possible future implications on hinge finding.

Sequence in the immediate neighborhood of a hinge was not found to be sufficient for substantive hinge prediction by a GOR-like method, although the latter is successful at predicting secondary structure. Similiarly, no particular sequential pairs of amino acid types were found to be overrepresented in hinges. However, we did find that combining amino acid propensity data with hinge propensities of active sites and secondary structure yielded some predictive information. The prediction method we present can easily be extended as additional hinge propensity data is reported. Indeed the publicly available Hinge Atlas can be used not only to obtain such data but also to test the resulting predictors. As an additional application, the Hinge Atlas can potentially be used to help find hinges by homology. We note, for instance, that a hinge occurring (unusually) in the helix connecting the two EF hands of calmodulin has also been found in the evolutionarily related Troponin C.

## Conclusion

We found that the amino acids glycine and serine are more likely to occur in hinges, whereas phenylalanine, alanine, valine, and leucine are less likely to occur. No evidence was found for sequence bias in hinges by a GOR-like method, nor for propensity towards sequential pairs of residues. Hinges tend to be small, but not hydrophobic or aliphatic. They are found less often in α-helices, and more often in turns or random coils. Active site residues were found to coincide significantly with hinges. Interestingly, however, the latter were not conserved. Lastly, hinges are also more likely to occur on the protein surface than in the core.

A consistent picture of hinge residues is suggested. In this view, hinges often occur near the active site, probably to participate in the bending motion needed for catalysis. They avoid regions of secondary structure. They are hypermutable, possibly due to the fact that they occur more often on the surface than in the core. These correlations yield insights into protein flexibility and the structure-function relationship. Strong sequence-based hinge prediction, however, remains a goal for future work.

## Authors' contributions

SF annotated hinge locations, performed the statistical studies and wrote the manuscript, web tools, and most of the algorithms. LL computed the evolutionary conservation of hinge and active site residues. NC ran FlexProt and generated graphics for all morphs in MolMovDB, and in other ways provided high performance supercomputing support to the hinge prediction project. MG supervised the project and edited the paper. All authors read and approved the final manuscript.
